# The effect of music interventions in autism spectrum disorder: a systematic review and meta-analysis

**DOI:** 10.3389/fnint.2025.1673618

**Published:** 2025-10-28

**Authors:** Laura Navarro, Nour El Zahraa Mallah, Wiktor Nowak, Jacobo Pardo-Seco, Alberto Gómez-Carballa, Sara Pischedda, Federico Martinón-Torres, Antonio Salas

**Affiliations:** ^1^Unidade de Xenética, Instituto de Ciencias Forenses, Facultade de Medicina, Universidade de Santiago de Compostela, and Genética de Poblaciones en Biomedicina (GenPoB) Research Group, Instituto de Investigación Sanitaria (IDIS), 15706 Hospital Clínico Universitario de Santiago (SERGAS), Santiago de Compostela, Galicia, Spain; ^2^Genetics, Vaccines and Infections Research Group (GenViP), Instituto de Investigación Sanitaria de Santiago, 15706 Universidade de Santiago de Compostela, Santiago de Compostela, Galicia, Spain; ^3^Centro de Investigación Biomédica en Red de Enfermedades Respiratorias (CIBER-ES), Madrid, Spain; ^4^Department of Pediatrics, Translational Pediatrics and Infectious Diseases, 15706 Hospital Clínico Universitario de Santiago de Compostela, Santiago de Compostela, Galicia, Spain

**Keywords:** autism spectrum disorder, neurodegeneration, neurodevelopment, music-based interventions, meta-analysis

## Abstract

**Introduction:**

Several disciplines have explored the relationship between autism spectrum disorder (ASD) and music, though most insights derive from cognitive sciences. This systematic review and meta-analysis synthesize evidence on the therapeutic effects of music-based interventions (MI) on communication, behavior, social engagement, attention, and quality of life in autistic individuals. It also examines how participants perceive and process music, situating therapeutic findings within this perceptual framework.

**Methods:**

From a total of 346 publications screened in PubMed, Cochrane Library, and WILEY Online Library databases, 120 were included, of which 15 met the criteria for quantitative evaluation and meta-analysis, to assess the state- of-the-art of research on music and autism in the fields of neuropsychology and cognitive sciences. The reviewed studies span a range of methodologies, including randomized controlled trials and qualitative research, and incorporate diverse MI strategies, such as active music-making, structured listening, and improvisational techniques.

**Results:**

Despite methodological heterogeneity, the findings suggest a moderate overall beneficial effect of MI, particularly in enhancing social interaction (z = 1.89, *p*-value = 0.06), verbal communication—especially vowel articulation (z = 2.93, *p*-value = 0.01), behavior (z = 1.92, *p*-value = 0.06; after outlier removal), and quality of life (z = 1.67, *p*-value = 0.09).

**Discussion:**

This study highlights music’s potential as a non-invasive, engaging therapeutic medium that elicits emotional, cognitive, and social responses in individuals on the spectrum. Given evidence of context-sensitive and domain-specific strengths in musical abilities, music emerges as a promising therapeutic approach. Future studies should investigate individual variability in response to MI, aim to standardize outcome measures, and assess long-term effects. Such efforts will support more personalized, neurodiversity-affirming therapeutic models in autism care.

## 1 Introduction

Autism Spectrum Disorder (ASD) is a complex neurodevelopmental condition characterized by altered perception (Tavassoli et al., 2016; Okamoto et al., 2018), sensory processing difficulties (Randell et al., 2019; Patil and Kaple, 2023), repetitive behaviors, rigid routines, a strong preference for sameness, an intense interest in specific topics or activities, and ongoing challenges in social communication (American Psychiatric Publishing, 2022). Autistic children often show a variety of unusual behaviors during their early years, compared to typically developing (TD) children, including resistance, repetitive actions, irritability, social withdrawal, reduced engagement, stereotyped behaviors, and atypical speech patterns. This might help explain why they struggle to integrate into society and build positive peer and family relationships, and why they often lack the social skills needed for everyday social situations.

ASD can profoundly affect both children and their parents, impacting daily life, finances, physical health, and mental well-being (Hossain et al., 2020). Due to the complex nature of ASD, which involves a mix of developmental and environmental factors and genetics, there is an ongoing debate about how the current treatments for ASD manage behavioral symptoms. Some interventions that have been used in the past include Applied Behavioral Analysis (ABA) (Voss et al., 2019; da Silva et al., 2023), Cognitive Behavioral Therapy (CBT) (Wood et al., 2020; You et al., 2024), sensory integration training (Randell et al., 2022), and pharmacological treatments (McCracken et al., 2002). However, these approaches often require a long treatment period, and since every autistic child is unique, the safety and effectiveness of these therapies are not yet fully supported by robust evidence (Sharma et al., 2018). Moreover, many of these interventions are limited in number and lack robust empirical validation. Therefore, it is crucial to identify scientifically sound and effective therapeutic approaches that can alleviate certain symptoms in autistic children and improve their behavioral outcomes.

Music-based interventions (MI), such as singing, playing instruments, music listening, and music therapy, are one of the therapeutic approaches that have been gaining interest over the past few decades, as a way to support autistic individuals (Chenausky and Schlaug, 2018; Remington and Fairnie, 2017; Cook et al., 2019). Such interventions are notable for their connection with people through verbal and non-verbal modalities. These approaches tap into the unique qualities of music to achieve a range of developmental goals, such as enhancing communication (He, 2022; Bieleninik et al., 2017), encouraging emotional expression (Brown, 2017; Quintin et al., 2011), improving social interaction (Bieleninik et al., 2017; Thompson, 2018), and boosting the overall Quality of Life (QoL) (Thompson, 2018) for autistic individuals. Consequently, these interventions, which are comparatively more accessible and cost-effective than typical pharmacological treatments, highlight music’s universal appeal and its unique capacity to engage multiple domains of functioning in autism, including cognitive, emotional, and social processes.

The benefits of music go beyond its fundamental neurological effects and play a significant role in neuro-rehabilitation practices (Pantev and Herholz, 2011). Neuroscience research has uncovered several links between music and autism, particularly highlighting the distinct cognitive and sensory processing traits found in autistic individuals (Kim et al., 2009; Wagener et al., 2021; Sharda et al., 2018; Lai et al., 2012). For instance, neuroimaging studies have revealed that music activates different brain regions related to auditory processing in autistic people compared to TD. A study (Sharda et al., 2018) utilizing functional Magnetic Resonance Imaging (fMRI) to compare how the brains of autistic and non-autistic individuals respond to music has indicated that those with autism exhibited greater activation in brain areas linked to music perception, such as the primary auditory cortex and regions associated with emotional processing. This suggests that music may have a more pronounced effect on brain networks in autistic individuals.

Although numerous studies (Geretsegger et al., 2022; Applewhite et al., 2022; Gao et al., 2024) have examined the effects of MI on various outcomes in autistic individuals, the results remain highly variable and at times contradictory. Recent systematic reviews, including Applewhite et al. (2022) that examined the impact of music on individuals with or at risk for autism, and the Cochrane review by Geretsegger et al. (2022), which assessed the efficacy of MT for autistic people, highlight substantial heterogeneity in study design, participant characteristics, intervention types, and outcome measures, resulting in inconsistent conclusions. To address this, our review aims to advance the field by (i) clarifying current evidence regarding the musical abilities and perceptual processing of autistic individuals (Section 3.1), thereby highlighting effective musical practices that contribute to a deeper understanding of music as a valuable tool in both everyday life and therapeutic contexts for this population, and (ii) conducting a meta-analysis of MI interventions (Section 3.2), with particular attention to outcomes such as social interaction, communication, QoL, attention, and behavioral regulation. In doing so, this work extends prior reviews by integrating perspectives on both the cognitive-musical profile of autistic individuals and the therapeutic potential of MI.

## 2 Methodology

### 2.1 Systematic review

Our investigation has a dual approach: (i) to investigate the relation between music and autism by analyzing the evidence regarding the musical processing of music of autistic individuals, and (ii) to provide a better understanding of how music influences different aspects of autism by integrating insights from neuropsychology.

A systematic review was conducted using PubMed, Cochrane Library, and WILEY Online Library databases, with the search extending between January 1st, 2000, and February 28th, 2024. To ensure comprehensive results relevant to autism, we only selected the search term “autism” based on its prevalence in literature. The term “music” was purposely kept broad to avoid restricting the scope of the study. We used the [tiab] field tag (title and abstract) to narrow the search to articles containing these specified terms, effectively filtering out irrelevant papers. Hence, the search query used was: (“autism”[tiab]) AND (“music”[tiab]).

The initial search yielded 346 publications, which were subsequently refined by eliminating duplicates, systematic reviews, and meta-analyses using the databases’ filtering tools. The selection was further narrowed to include only English-language articles focused on human subjects, published between 2000 and 2024, to ensure the inclusion of recent and relevant studies. This process resulted in a total of 285 publications. Of these, 65 were excluded for being reviews or systematic analyses. Following a detailed evaluation of the remaining 220 articles, additional exclusions were made based on predefined inclusion and exclusion criteria. Studies were excluded if they: (a) involved mixed therapeutic approaches incorporating music alongside other interventions, as these could confound the isolated effects of MI; or (b) did not focus on autism as the primary population of interest. These criteria were established to guarantee conceptual clarity and to enhance the validity of the synthesis, following best-practice recommendations for systematic reviews as outlined by Shea et al. (2017). After this thorough analysis, 115 publications met all inclusion criteria and were selected for the systematic review.

**Figure 1:**
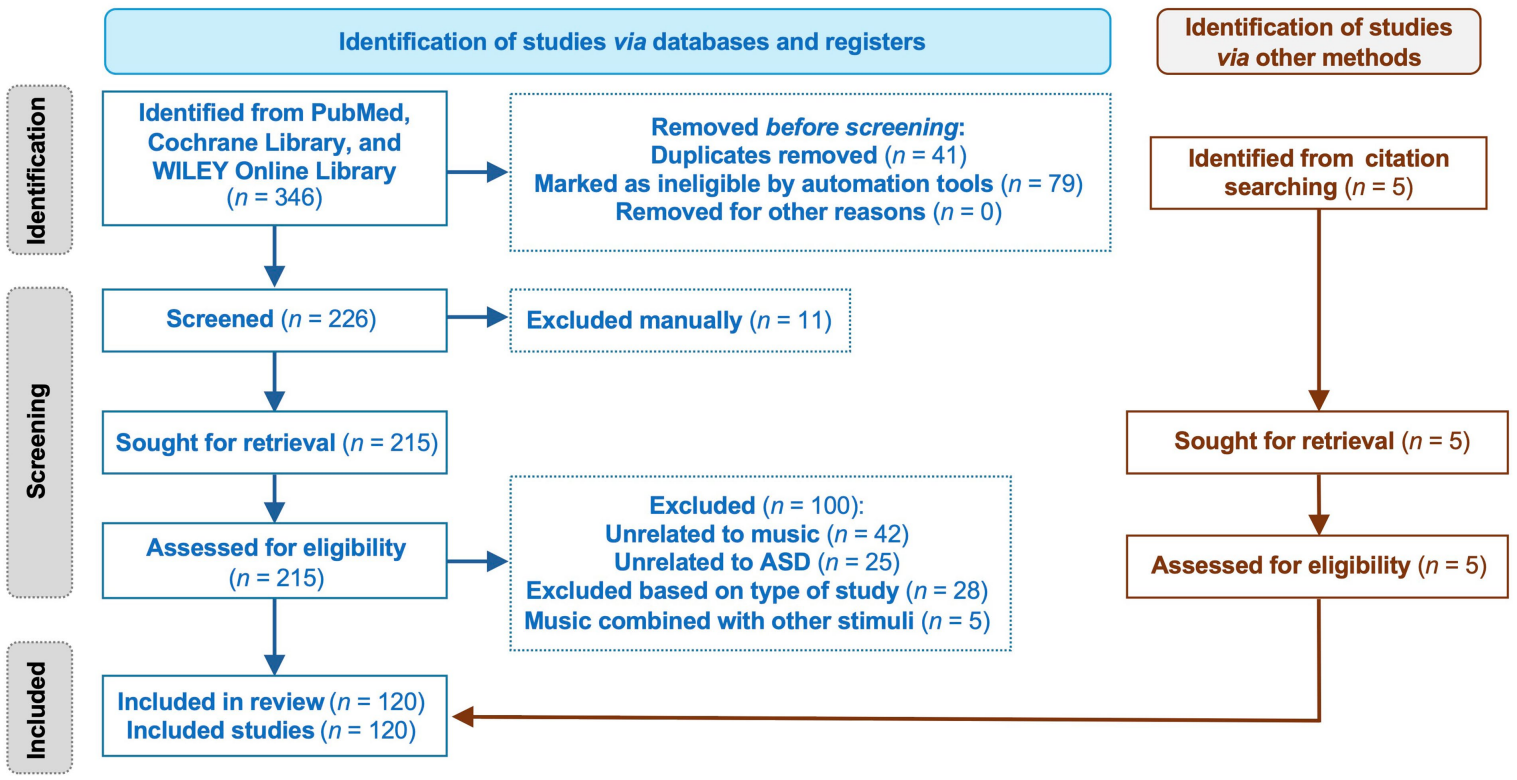
Flowchart of the study selection process following PRISMA guidelines. This visual representation illustrates how studies were identified, screened, assessed for eligibility, and ultimately included. The diagram details records retrieved via PubMed and citation tracking, as well as the number of studies excluded at each stage and the reasons for their exclusion. The structure follows the updated PRISMA model as outlined by Page et al. (2021).

Additionally, a total of five additional relevant articles were manually added to the list by scrutinizing the reference list of the selected articles. With a total of 120 publications, the findings were categorized into two main categories: (i) the synthesis of musical processing in autistic individuals and (ii) the effects of MI for autistic individuals. The present review was undertaken and is reported following the Preferred Reporting Items for Systematic reviews and Meta-Analyses (PRISMA) guidelines (Figure 1).^1^

### 2.2 Meta-analysis

In addition to the systematic review and qualitative synthesis, this study includes a meta-analysis of 15 studies that met the criteria for quantitative evaluation, focusing on primary outcome domains commonly examined in autism research. A structured and standardized methodology was employed to integrate findings across these outcome categories. Two reviewers independently extracted the relevant data, and discrepancies were resolved by consensus. Studies were excluded if they lacked a control group, exclusively reported short-term physiological markers, or failed to isolate the effect of MI from other co-occurring therapies. Data extracted from each study included sample sizes, means, and standard deviations for intervention and control groups, outcome measures used, intervention duration, and design features. Pooled mean differences and 95% confidence intervals (CIs) for pre-to-post changes were calculated for each domain, comparing MI groups with non-music controls. A random-effects model was applied to account for heterogeneity among studies, using the DerSimonian and Laird method (DerSimonian and Laird, 1986). Random-effects models are generally preferred when between-study heterogeneity is non-negligible, as was the case in several domains of this review, with I^2^ values exceeding 50% (Balduzzi et al., 2019). Although this approach typically results in wider CIs due to the inclusion of between-study variance, it provides more conservative and generalizable estimates under conditions of heterogeneity. The diversity of intervention formats and participant profiles justified this modeling choice, which supports the broader applicability of the findings. Heterogeneity was assessed through the I^2^ statistic, τ^2^ (tau-squared), and Cochran’s Q test. In cases of substantial heterogeneity, subgroup analyses were conducted to identify potential sources, and statistical significance between subgroups was tested using chi-squared (χ^2^) comparisons. Forest plots were generated to visualize individual and combined effect sizes. Although the number of studies included was insufficient for formal publication bias testing (e.g., funnel plots or Egger’s test), sensitivity analyses were performed to evaluate the stability of results by excluding studies with extreme values or high risk of bias. Subgroup analyses were also stratified by specific measurement instruments (e.g., ADOS-SA, SRS, ASSP), recognizing the varied constructs captured by each outcome assessment. To estimate the potential variability of treatment effects in future research settings, prediction intervals were computed, considering both intra- and inter-study variance. One recurring challenge in autism meta-analyses is the lack of reported standard deviations for change-from-baseline outcomes (Pearson and Smart, 2018). In accordance with Cochrane recommendations (Higgins and Green, 2011), missing SDs were imputed using a conservative correlation coefficient of *r* = 0.8, consistent with assumptions made in prior meta-analytic work (Pearson and Smart, 2018; Giuliano et al., 2017). To standardize treatment effects, outcome directions were harmonized by inverting scores where necessary. In cases where different tools assessed the same domain, scores were transformed to a common scale to support comparability. Nevertheless, variability in scoring systems and measurement units meant that not all studies could be integrated into the same subgroup analysis, limiting some within-domain comparisons. Despite this, the overall consistency of results supports the robustness of the findings.

Separate meta-analyses were conducted for each major domain (e.g., communication, social engagement), ensuring interpretability and consistency of effect sizes.

All statistical analyses were conducted using the R meta package (Balduzzi et al., 2019), with forest plots illustrating the results and the statistical significance of pooled effects assessed using z-tests.

## 3 Results

Based on the included studies’ results and our investigation’s goal, the studies were divided into two main categories: (i) synthesis of current research on musical processing in autistic individuals, focusing on their perception and musical abilities and (ii) discovery of benefits and therapeutic effects of music for autistic individuals.

### 3.1 Musical processing and musical abilities in autistic individuals. A musical phenotype in autism?

We analyzed 45 articles on how music’s structural aspects are processed in populations with autism (overall musically untrained), compared to TD children. We aimed at investigating whether specificities exist in the musical brains of autistic individuals, especially in musical perception and emotion processing. The main analyzed categories were pitch processing (pitch discrimination and absolute pitch), auditory pleasantness, emotional processing, other musical domains, and the function of music in their lives.

#### 3.1.1 Pitch discrimination abilities

The ability to identify a specific pitch involves cognitive processes such as auditory perception and memory. In this section, we examined the nature of auditory mechanisms underlying musical recognition and assessed whether autistic individuals exhibit distinct abilities or processing patterns. This field is the main corpus of papers corresponding to musical abilities in autism.

The main finding is the special sensitivity of autistic individuals to pitch changes and their enhanced ability to discriminate musical parameters, providing evidence for preserved, or even superior, pitch processing in this population (Bonnel et al., 2003; Heaton, 2003; Heaton, 2005; Jävinen-Pasley and Heaton, 2007; DePape et al., 2012; Chowdhury et al., 2017; Heaton et al., 2018; Wang et al., 2022; Germain et al., 2019). According to Chowdhury et al. (2017), auditory perception is related to non-verbal reasoning rather than verbal abilities in autism and TD. Some studies connect autism’s ability to recognize different pitches to superior memory, including long-term melodic memory (Heaton, 2003; Stanutz et al., 2014) or working memory, and focused attention (Bennett and Heaton, 2012). However, Heaton et al. (2018) affirmed that autistic individuals perform better than TD controls on pitch identification despite being impaired in short-term memory. Some studies detect similar recognition of melodic contour in autism and TD (Mottron et al., 2000), and other research affirmed that auditory imagery is lower in autism than in TD controls (Bacon et al., 2020).

Various studies compared the effect of listening to music with listening to speech. Jävinen-Pasley and Heaton (2007) and Heaton et al. (2007) demonstrated that speech inclusion in perception activities declines the levels of accuracy in pitch discrimination of autistic individuals. Similarly, DePape et al. (2012) demonstrated that autistic individuals are more impaired in speech tasks than in music tasks. In this line, Lai et al. (2012) compared the neural systems and showed that autistic individuals activated the left inferior frontal gyrus more than controls in song stimulation; however, the opposite happened with speech stimulation. Sharda et al. (2015) found that functional frontotemporal connectivity was preserved during sung-word listening in autism in contrast to speech-word, enhancing the importance of MI with this population. Other arguments present evidence of how autism mental representations of pitch contours could be across domains, and implications for using music to improve language (Wang et al., 2023).

#### 3.1.2 Absolute pitch

Absolute pitch (AP) is an extreme phenotype associated with naming or producing a musical tone without any reference. Several researchers have suggested that AP is a normally distributed complex trait with a strong genetic component (Baharloo et al., 1998; Gregersen et al., 1999), with a prevalence of <1% in the general population (Profita and Bidder, 1988).

The outstanding study of DePape et al. (2012) measured the prevalence of AP processing in autism using a task that does not require explicit knowledge of musical structure, which can be used by non-musicians with and without autism, estimating a prevalence of 11% in autism, which is remarkable compared to 1 in 10,000 in typical populations.

Autism’s neurocognitive theories might explain this co-occurrence, while some case studies illustrate evidence of AP in autistic individuals who possess extraordinary musical abilities (Heaton et al., 2008; Brenton et al., 2008; Bouvet et al., 2014; Courchesne et al., 2020). Research involving musicians has found autism features in those with AP, suggesting a link between the two conditions (Brown et al., 2003; Dohn et al., 2012; Wenhart et al., 2019a; Wenhart and Altenmuller, 2019; Wenhart et al., 2019b) and highlighting the detail-oriented cognitive style, imagination, perceptual functioning, and hyper-systemizing present in both. A noteworthy study emphasizes a potential connection between autism traits, brain connectivity, and AP ability (Wenhart et al., 2019b), suggesting a less efficient and less small-world-structured functional network in AP, consistent with findings from autism research.

Curiously, one study focusing on pitch production reveals a vocal imitation deficit in autistic individuals that is specific to AP, but not to relative pitch, across both speech and music domains (Wang et al., 2021). This may be related to the fact that autistic individuals often exhibit atypical imitation of actions and gestures.

#### 3.1.3 Other musical abilities

Pitch recognition is related to the ability to produce a determined sound properly. The processing involving human listening, specifically musical discrimination, is inevitably joined to musical production through intonation (Morrison and Fyk, 2002). Regarding the auditory processing that involves pitch production through voice, Wang et al. (2022) found that imitating musical intonation is intact in autistic individuals. Other studies compared this ability of pitch production in musical language with speech production, demonstrating the preservation of pitch production and a prosodic impairment (Jiang et al., 2015; DePriest et al., 2017).

According to Heaton (2003), abilities such as pitch memory and labeling are superior in autism and may facilitate performance in harmonic contexts for autistic individuals. In a subsequent study, Heaton et al. (2007) affirmed that the most striking finding was the absence of significant differences in performance patterns between autism and control participants; both groups were similarly influenced by harmonic context in their perception. DePape et al. (2012) found that musical processing is relatively preserved in autism in many aspects: pitch detection, pitch memory, harmonic and metrical processing. Other minor examinations focused on rhythmic perception and affirmed that the tempo of acuity was preserved (Heaton et al., 2018; Dahary et al., 2024).

#### 3.1.4 Auditory pleasantness

Auditory perception and overall musical pleasantness depend on both the acoustic properties of the stimulus and the cultural experiences that shape individual musical preferences. Six studies have investigated musical preferences in autistic individuals, while others have focused on the benefits of different types of music and the importance of selecting appropriate musical content for therapeutic purposes. While recent research (Michel et al., 2024) shows autistic adults rated instrumental sounds more pleasant than vocal sounds, Kalas (2012) discovered the effectiveness of joint attention of simple music (short melody without syncopation or chromatism, accompanied by I-IV-V chords) for severe autism, in contrast to the function of complex music for mild/moderate autistic individuals. In qualitative research (Sravanti et al., 2023), parents of autistic children reported an active response to music and a preference for rhythmic music. All research compared the auditory pleasantness of autistic individuals with controls, reaching relevant results as autism are more sensitive to consonance and dissonance, appreciating a larger variety of music, from Mozart to Schoenberg (Masataka, 2017). The most compelling study examines functional brain connectivity between specific regions while comparing familiar and unfamiliar songs (Freitas et al., 2022), concluding that there is no difference in how autistic and TD individuals process familiar music. However, this author found significant differences in how the autistic brain processes unfamiliar music compared to the TD, activating the alpha band and increasing connectivity. Also, in this research, autistic children showed no difference in the magnetoencephalography of familiar and unfamiliar music. However, Lanovaz et al. (2012) found that preferred music reduced vocal stereotypy in four autistic children.

#### 3.1.5 Emotional processing of music

Lately, processing musical emotions in autistic individuals, such as emotion recognition or emotional expressions, has gained attention. The impairment to identify, recognize, or verbalize emotions is known as alexithymia, but autistic individuals could be affected only in the ability to verbalize or articulate the expression of emotions (Allen et al., 2009; Allen et al., 2013). Music improves emotion recognition in autistic individuals (Wagener et al., 2021; Redondo Pedregal and Heaton, 2021), but many studies contribute deeply to understanding the mechanisms underlying emotional processing.

Some studies demonstrated the physiological effect of music on the autistic population by measuring the emotional response through skin conductance or the autonomic nervous system’s reaction, or evaluating the capability to recognize emotions, demonstrating no notable difference between autism and controls (Quintin et al., 2011; Allen et al., 2013; Whipple et al., 2015; Jarvinen et al., 2016; Leung et al., 2023), and the accuracy in emotional recognition in autistic individuals (Whipple et al., 2015; Leung et al., 2023; Sivathasan et al., 2023).

Moreover, using fMRI, neuroscientific studies found similar neural networks during musical processing in emotion recognition in autism and TD (Caria et al., 2011; Gebauer et al., 2014), considering music as a domain of preserved ability. However, Caria et al. (2011) associated the strong emotional response to happy music in autism with decreased activity in the cerebellum and premotor areas, showing a possibly altered rhythm perception.

However, some studies found an impairment in judging the emotional expressivity of music (Bhatara et al., 2010) or established differences between autism and TD in the emotional response to music. Quintin et al. (2011) studied the recognition of happiness, sadness, scariness, and peacefulness through 20 musical clips, and they only found a difference between autistic and TD adolescents in recognition of the peaceful music, which was the most difficult. Gebauer et al. (2014) showed that autistic individuals demonstrate more arousal activity and cognitive load in happy than in sad music, unlike TD controls.

Leung et al. (2023) found that the processing speed of emotions through music was slower in the autistic group compared to the neurotypical (NT) group, suggesting that autistic individuals may employ different emotion-processing strategies. Furthermore, Wagener et al. (2021) reported higher reaction times in autism. Stephenson et al. (2016) found reduced skin conductance in response to music in autistic adolescents, as well as a decrease in physiological responsiveness with age, contrary to NT controls.

#### 3.1.6 Qualitative role of music

Several studies employing qualitative or mixed-method designs have explored the meanings and functions of music in the lives of autistic individuals. To illustrate, Kirby and Burland (2021) identified four primary roles that music plays: emotional, cognitive, identity-related, and social. Notably, they found these roles to be consistent across autistic and TD individuals. Importantly, autistic adults often use music for emotional self-regulation, mood modulation, and emotional states management (Allen et al., 2009; Korošec et al., 2024; Lense et al., 2022). Furthermore, music also serves as a means of fostering social connection and interaction (Korošec et al., 2024; Lense et al., 2022), highlighting its potential as a bridge for communication and social engagement in autistic populations.

### 3.2 Music-based interventions’ (MI) impact on ASD

MI can play a crucial role in improving people’s lives, demonstrating the potential of music as a valuable therapeutic tool for supporting autistic individuals. In the 75 studies included in this systematic review, 34 utilized a Music Therapy (MT) approach and 41 a different MI, of which 17 are based on music listening (ML), 7 on playing musical instruments, 6 on singing and creating songs, 5 on music and movement intervention, and 6 in combining musical activities. All of the musical interventions reported benefits, but especially an active engagement, such as MT, playing instruments, music and movement or a combination of playing, singing and improvisation can effectively minimize core symptoms in autistic children and can help in reducing autism severity, enhancing social engagement, and decreasing repetitive behaviors, among others (Ren et al., 2022).

Many different techniques of MT have been utilized in the 34 studies reviewed, such as Receptive Music Therapy, Improvisational Music Therapy, or Neurological Music Therapy, among others, impacting especially in social engagement and behavioral outcomes [see for example, LaGasse (2014)]. In a comparative study, Rabeyron et al. (2020) found that MT led to greater clinical improvements, measured using the Clinical Global Impression (CGI) scale, and greater reductions in stereotypical behaviors than ML alone. Notably, MT was associated with enhanced social engagement in 27 studies, whereas other MI reported similar benefits in 22 studies. Behavioral improvements were observed across various musical activities, with 11 MT studies and 9 ML interventions specifically highlighting such effects.

Next, we summarize the findings of the 15 studies that fulfilled the inclusion criteria for our meta-analysis, which are drawn from the larger pool of 120 studies identified in the review. To provide context, we also highlight relevant results from the broader review at the beginning of each outcome section, including a narrative on verbal and non-verbal communication, attention, behavior, QoL, and social interaction. Table 1 presents the outcomes and measures for the included studies.

**Table 1:** Overview of studies included in the meta-analysis, including intervention type, duration, comparators, and outcome assessment tools.

Reference	Participants	Age	Intervention	Duration	Comparators	Outcomes	Measures
Bieleninik et al. (2017)	EG: 182 CG: 182	4–7	Improvisational music therapy	5 m	SC	GI/QoL/SI	Autism Diagnostic Observation Schedule-Social Affect (ADOS-SA) Social Responsiveness Scale (SRS) Total ADOS-Restricted and Repetitive Behaviors (ADOS-RRB) Parent-reported QoL of the child (QoL-child) and of the family as a whole (QoL-family)
Chenausky et al. (2022)	EG: 8 CG: 6	5–11	Singing and drumming	25 s	PA	VC	% Vowels Correct (%Vc) % Syllables Approximately Correct (%Sac) % Consonants Correct (%Cc)
Gattino et al. (2011)	EG: 12 CG: 12	7–12	Singing, playing instruments, and movement to music	7 m	SC	G/nVC	Childhood Autism Rating Scale, Brazilian version (CARS-BR) CARS-BR non-verbal communication domain CARS-BR verbal communication domain
Ghasemtabar et al. (2015)	EG: 13 CG: 14	7–12	Music listening, playing instruments, singing, and dancing	45 d	SC	SI	Social Skills Rating System Scale (SSRS)
Kim et al. (2008)	EG: 5 CG: 5	3–5	Improvisational music therapy	12 w	PA	GI/SI	Social Approach Subscale (Pervasive Developmental Disorder Behavior Inventory, PDDBI) Early Social Communication Scale (ESCS)
LaGasse (2014)	EG: 9 CG: 8	6–9	Music-based activities	5 w	PA	GI/nVC/A	Social Responsiveness Scale (SRS) Total Autism Treatment Evaluation Checklist (ATEC) Total
LaGasse et al. (2019)	EG: 7 CG: 7	5–12	Playing instruments	5 w	SC	B	Behavior measures
Liu et al. (2025)	EG: 18 CG: 14	3–5	Watching musical videos	3 d	PA	VC	Verbal Production Evaluation Scale (VPES)
Ren et al. (2022)	EG: 15 CG: 15	5–8	Listening to music and dancing	3 m	SC	B/GI	Childhood Autism Rating Scale (CARS) Autism Treatment Evaluation Checklist (ATEC)
Sa (2020)	EG: 11 CG: 12	10–14	Music listening and playing instruments	6 w	SC	A	Test of Everyday Attention for Children 2 (TEA-Ch 2)
Schwartzberg and Silverman (2016)	EG: 16 CG: 14	9–21	Singing and playing instruments	3 d	PA	GI/SI/VC	Autism Social Skills Profile (ASSP) Comprehension checks (CCs)”
Sharda et al. (2018)	EG: 26 CG: 24	6–12	Involved use of improvisational approaches through song and rhythm	8–12 w	PA	B/nVC/QoL/SI/VC	Social Responsiveness Scale (SRS-2) Beach Center Family Quality of Life Scale (FQoL) Children’s Communication Checklist (CCC-2) Peabody Picture Vocabulary Test-4 (PPVT-4) Vineland Adaptive Behavior Scales (VABS)
Sivathasan et al. (2023)	EG: 12 CG: 12	5–12	Rhythm-based interventions	10 w	SC	B	Repetitive Behavior Scale (RBS)
Thompson et al. (2014)	EG: 11 CG: 10	3–6	Singing and movement to music	16 w	SC	GI/SI/VC	Parent–Child Relationship Inventory (PCRI) Vineland Social Emotional Early Childhood Scales (Vineland SEEC) Social Responsiveness Scale-Preschool version (SRS-PS) MacArthur-Bates Communicative Development Inventories-Words and Gestures (MBCDI-W&G)
Williams et al. (2024)	EG: 11 CG: 8	2–5	Listening and singing engaged with physical activities	18 w	PA	B/SI/VC	Social Responsiveness Scale (SRS-2) Expressive One-Word Picture Vocabulary Test (EOWPVT-4) Receptive One-Word Picture Vocabulary Test (ROWPVT-4) MacArthur-Bates Communicative Development Inventories (CDI) Vineland Adaptive Behavior Scales–third edition (VABS-3)”

Age expressed in years. EG: experimental group; CG: control group; m: months; d: days; s: sessions; w: weeks; SC: Standard Care; PA: ‘Placebo’ activity without music; A: Attention; B: Behavior; GI: Global Improvement; nVC: Non-verbal Communications; QoL: Quality of Life; SI: Social Interaction; VC: Verbal Communication.

#### 3.2.1 Attention

Autism is characterized by differences in attention regulation, which can influence cognitive processing and sensory perception. Music has emerged as a potential tool to manage attention in autistic people. It engages both auditory and emotional networks, which can lead to better focus and less distractibility. A study (Romer et al., 2024) examined how young autistic and non-autistic adult drivers performed while listening to music in various situations, and demonstrated that music might have distinct effects on attention and task performance in autistic individuals compared to TD. Additionally, a study of a single-case experimental multiple baseline design (Vaiouli et al., 2015) investigated how parent–child collaborative MT could benefit autistic children and their mothers, indicating potential improvements in attention for children as well as improved maternal well-being. Moreover, Kim et al. (2008) reported that autistic children who engaged with music demonstrated better attention than those who did not. Also, Pasiali et al. (2014) highlighted the potential of MI in advancing attention in autistic individuals, emphasizing how engaging and motivating music can be. Collectively, these studies highlight the significant role of music in enhancing attention in autism, suggesting that personalized MI might be helpful in both therapeutic and everyday contexts.

Structured MI, like rhythmic entrainment and melodic stimuli, might enhance attentional control and improve task performance in autism population as well. LaGasse (2014) provided evidence that rhythmic training through music can improve joint attention between autistic children. Furthermore, Sa (2020) supported these findings by showing that structured musical activities can facilitate attentional shifts and reduce distractibility in those with autism.

**Figure 2:**
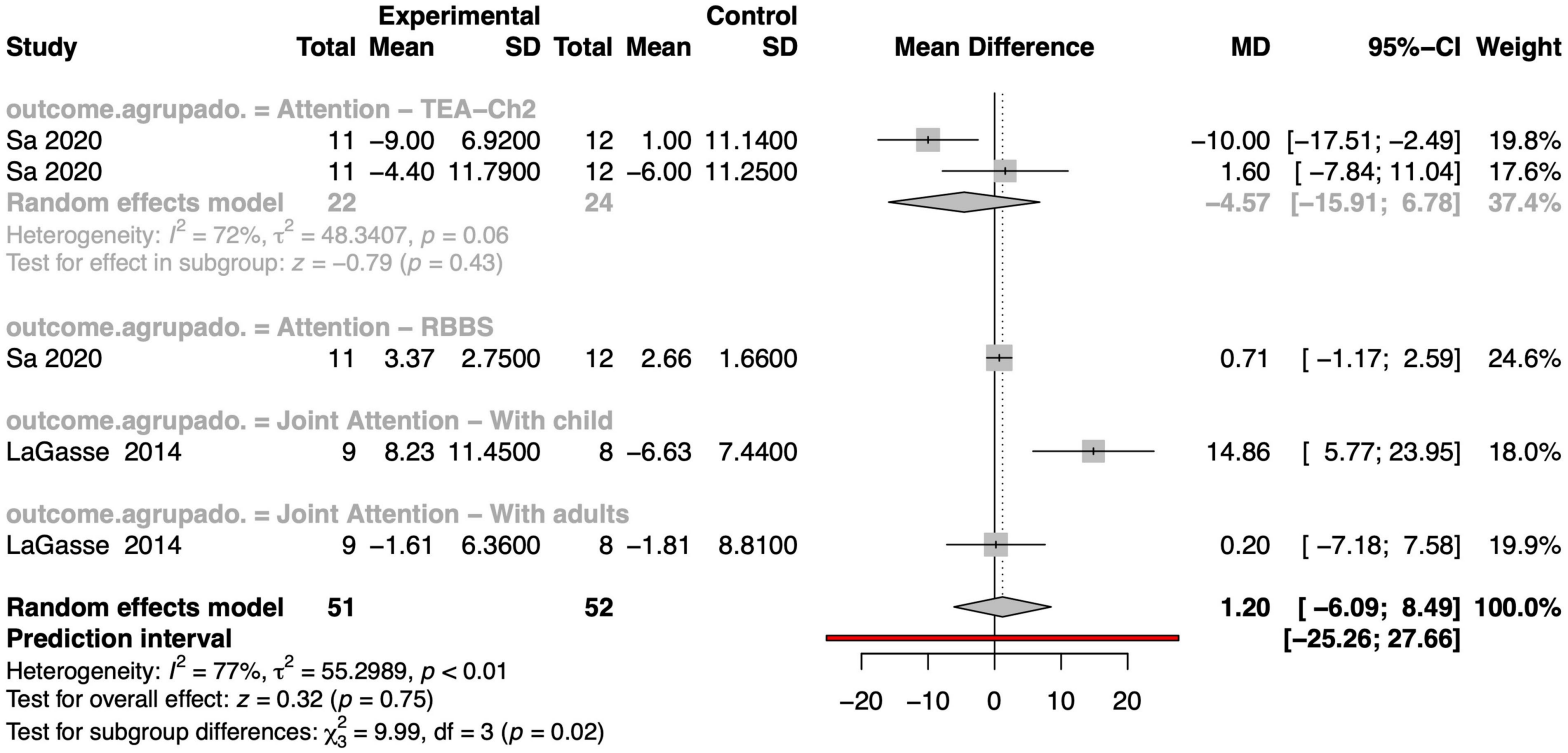
Forest plot illustrating the effect sizes (mean differences) for studies comparing interventions and controls about attention outcomes in autistic individuals. The plot includes each study’s mean difference (MD), 95% confidence interval (95% CI), and statistical weight. Summary estimates were calculated using a random effects model. Heterogeneity statistics (I^2^, τ^2^, and *p*-values), prediction intervals, and tests for subgroup and overall effects are also provided.

We conducted a meta-analysis to evaluate the impact of MI on attention in autistic individuals, using the Test of Everyday Attention for Children (TEA-Ch), the Red & Blues, Bags & Shoes (RBBS), and Joint Attention tests as primary outcome measures (Figure 2). The analysis included two methodologically sound studies (Sa, 2020; LaGasse et al., 2019) and employed a random-effects model to pool their results. The overall mean difference was 1.2 with a 95% confidence interval (CI) of [−6.09; 8.49], but the test for overall effect was not statistically significant (z = 0.32, *p*-value = 0.75). Heterogeneity was substantial (I^2^ = 77%, τ^2^ = 55.30, *p*-value < 0.01), indicating considerable variation in findings across studies. The studies in this meta-analysis contributed to comparable statistical weights, ranging from 17.6 to 24.6%. It is important to note the outlier behavior observed in one of the measurements reported by Sa (2020) (Figure 2). When excluding this outlier from the meta-analysis, the overall metrics improve notably (Supplementary Figure S1). Specifically, the pooled mean difference (MD) increases to 3.66 (95% CI: −2.49 to 9.80). Although the overall effect remains statistically non-significant, the test statistic shows a stronger trend (z = 1.17, *p*-value = 0.24). Additionally, heterogeneity decreases substantially (I^2^ = 67%, τ^2^ = 26.60, *p*-value = 0.03), though considerable variability across studies still persists.

Although the overall effect was not statistically significant, the MD of 1.2 suggests a positive trend favoring MI. However, the wide confidence interval and lack of statistical significance indicate that this trend should be interpreted with caution. Therefore, given the limited number of studies and small sample sizes, further research is necessary to clarify the potential of music to improve attentional functioning in this population, particularly concerning specific subtypes of attention.

#### 3.2.2 Behavior

Autism is marked by unique differences in how individuals process sensory information and behave, often showing repetitive patterns and difficulty with managing emotions. Music has been widely explored as an intervention to support behavioral and emotional growth in autistic individuals. Research suggests that MI can boost social engagement, ease anxiety, and improve adaptive behaviors by taking advantage of the structured, predictable, and emotionally expressive nature of music.

Recent studies have investigated how these MI affect behavior in autistic individuals. Williams et al. (2024) examined the use of MT by autistic adolescents and reported improvements in social interactions and a drop in anxiety levels. Moreover, Tahmazian et al. (2023) investigated how rhythmic auditory stimulation impacts repetitive behaviors in autistic children and showed a notable decrease in these behaviors following the intervention. Another piece of research from Liu et al. (2025) examined how musical activities can help autistic adults manage their emotions. It turned out that regularly participating in music-based activities improved emotional control and reduced aggressive behaviors.

Expanding upon the review, Lundqvist et al. (2019) investigated the impact of vibracoustic music on social behaviors in autistic adults and developmental disabilities, where the participants received sessions of low-frequency sound vibrations delivered through a vibroacoustic chair, combined with music. Notably, vibroacoustic music was found to reduce challenging behaviors and lower the incidence of self-injurious actions and stereotyped behaviors. These results highlight the potential of vibroacoustic music as a calming intervention to reduce behavioral challenges in autistic adults and related developmental disabilities. Overall, these insights support the notion that MI may positively influence various behavioral aspects in autistic individuals, including repetitive behaviors, social skills, anxiety, and emotional regulation.

**Figure 3:**
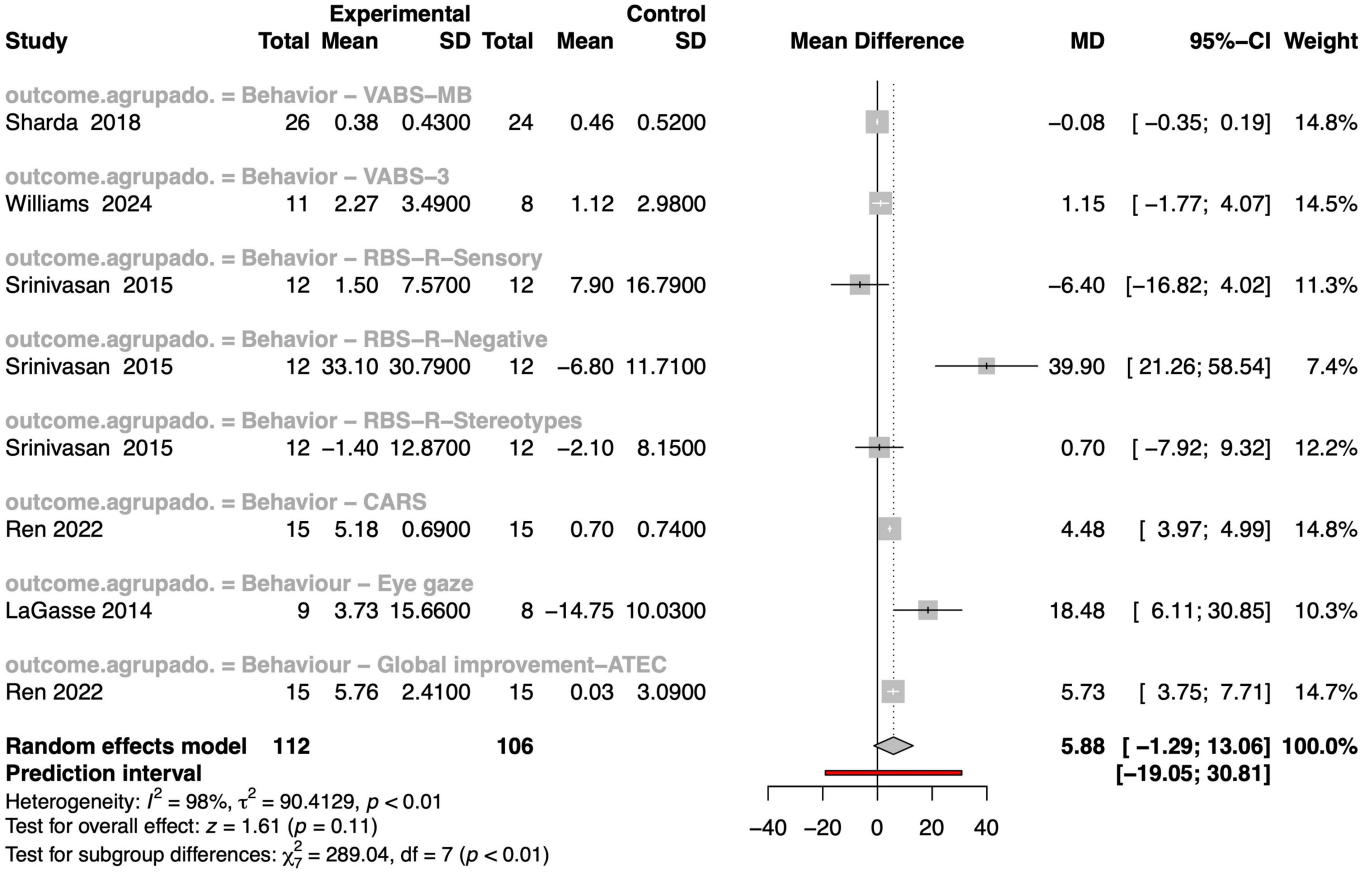
Forest plot depicting comparative mean differences for behavioral outcomes between intervention and control groups in autistic individuals. The plot presents mean differences, 95% confidence intervals, and weights for each study. Summary estimates were calculated using a random effects model, along with heterogeneity measures (I^2^, τ^2^, and *p*-values), prediction intervals, and subgroup/overall test results.

In the meta-analysis of behavioral outcomes, only five studies were included, with pooling data across studies using a random-effects model (Figure 3). The analysis incorporated several behavioral assessment tools, such as the Childhood Autism Rating Scale (CARS), Vineland Adaptive Behavior Scales (VABS), and Repetitive Behavior Scale (RBS). The overall test for effect yielded a MD that was not statistically significant (z = 1.61, *p*-value = 0.11), suggesting that the impact of music on general behavior, as currently measured, remains inconclusive. Importantly, heterogeneity across studies was extremely high (I^2^ = 98%, τ^2^ = 90.41, *p*-value < 0.01), indicating considerable variability in outcomes, populations, intervention types, and measurement instruments. Subgroup analyses revealed significant differences across behavioral scales (χ^2^ = 289.04, df = 7, *p*-value < 0.01), emphasizing that the magnitude and direction of effects may depend heavily on the specific behavioral domain assessed and the tool used. While some individual studies showed large positive effects (e.g., Williams et al., 2024), others reported mixed or null results, reflecting the complexity of measuring behavioral change in autism through MI.

Eliminating the outlier outcome represented by the RBS-R-Sensory measure from Srinivasan et al. (2015) significantly improves the overall metrics (Supplementary Figure S2). The effect estimate becomes more statistically robust, reaching marginal significance (z = 1.92, *p*-value = 0.06), suggesting a potential positive impact of music on general behavior. However, heterogeneity across studies remains extremely high (I^2^ = 98%, τ^2^ = 89.74, *p*-value < 0.01), indicating substantial variability in study results.

This meta-analysis provides suggestive statistical evidence of behavioral improvements. The observed trends and scale-specific heterogeneity indicate that MI may benefit certain behavioral aspects in autistic individuals. These findings warrant further investigation using standardized protocols, clearly defined behavioral outcomes, and larger sample sizes.

#### 3.2.3 Communication

Communication challenges have always been considered a fundamental feature of autism, yet there is a vast range of differences in how autistic individuals communicate and express themselves. This variation not only highlights the unique nature of the condition but also the complexity of communication itself. It involves not just the chosen words and their sequence, but also eye contact, facial expressions, gestures, and other nonverbal signals. Because of this complexity, music has gained attention as a potential means to enhance communication for those with autism. It taps into shared neural pathways that are crucial for emotional processing and social engagement. Studies suggest that MI, such as improvisational MT (Kim et al., 2009; Kim et al., 2008; Porter et al., 2017) and rhythmic entrainment (LaGasse, 2014; Srinivasan et al., 2015; Yoo and Kim, 2018), can enhance expressive abilities in autistic individuals. Moreover, the structured and predictable nature of music can create a comforting environment for communication, reduce anxiety, and encourage participation.

Another important connection between autism and music lies in language and verbal communication. Many autistic individuals face language challenges, such as delayed speech and unusual language development (American Psychiatric Publishing, 2022). Music, especially its rhythmic and melodic elements, has been found to aid in language and development (Bieleninik et al., 2017; Crawford et al., 2017). The rhythmic patterns found in music reflect the natural rhythms of speech, which can improve vocabulary and sentence structure in autistic children, making the learning process more effective and enjoyable.

Based on the previously mentioned findings, both verbal and non-verbal communications were examined in our investigation.

##### 3.2.3.1 Verbal communication

Autism affects cognitive processing and communication, often impacting learning styles and sensory experiences. Music is widely acknowledged as a powerful tool for autistic individuals, offering a structured yet adaptable medium that supports cognitive, emotional, and social development. Research suggests that music enhances learning by improving attention, memory, and language skills while reducing anxiety and sensory overload (Kim et al., 2008; Kaplan and Steele, 2005). The rhythmic and repetitive structure of music appears to align particularly well with the cognitive and sensory profiles of individuals on the autism spectrum, enhancing both engagement and comprehension (Latif et al., 2021; Boso et al., 2007). Furthermore, MI, such as MT and adaptive music education, has shown success in fostering communication and self-expression.

The research by Schwartzberg and Silverman (2016) may inspire music therapists and educators to integrate music-based stories into reading programs to improve comprehension skills in autistic children. Although this study did not reveal significant differences between the singing and reading groups, it highlighted the potential of MI to aid reading comprehension in autistic children. Furthermore, the findings emphasized the necessity of employing diverse instructional methods for teaching reading comprehension, suggesting that music can be a valuable element of a comprehensive educational strategy for autistic children.

Various studies highlighted the effectiveness of music-based approaches in both therapeutic and diagnostic contexts for autistic individuals (Cibrian et al., 2020; Bergmann et al., 2021) and have noted that MI not only improves motor skills in autistic children but also serves as a diagnostic tool, especially for populations with intellectual disabilities. Therefore, integrating music into learning environments can yield fruitful benefits for autistic individuals, fostering their development and overall well-being.

**Figure 4:**
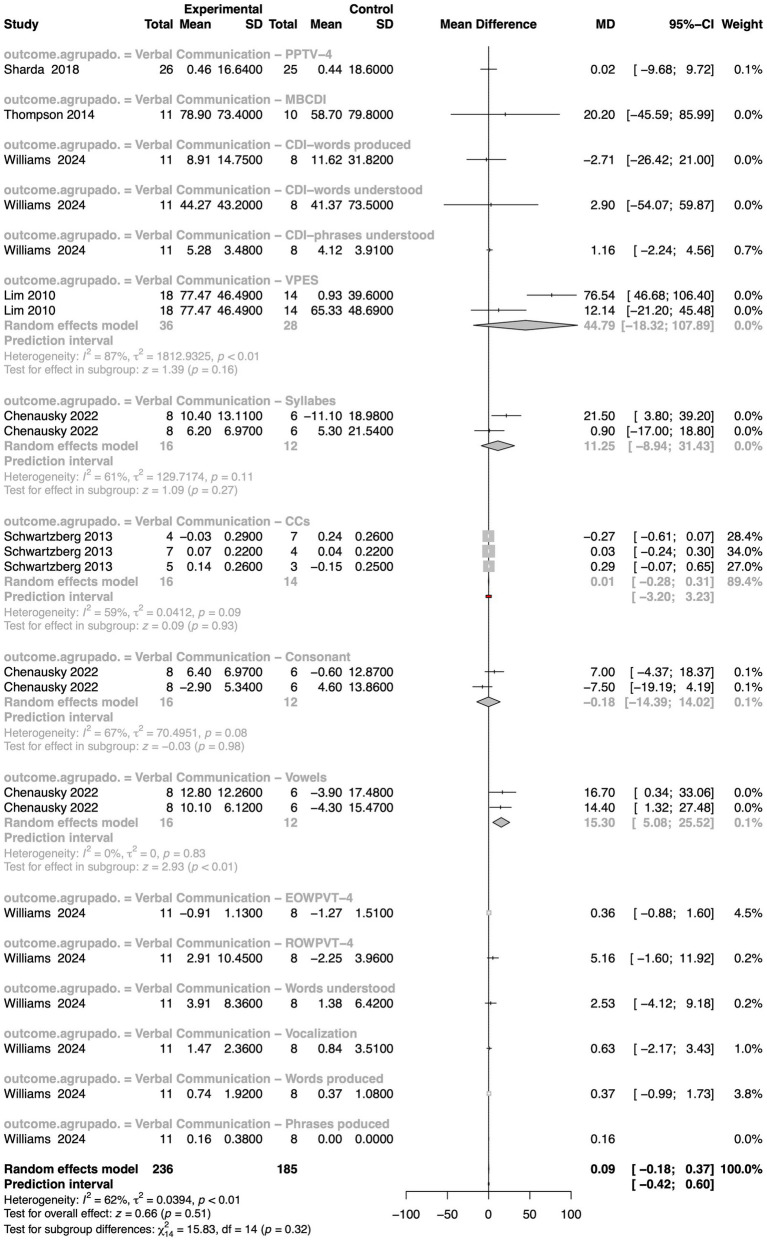
Forest plot summarizing intervention effects on verbal communication in autistic individuals. Individual study data include mean differences, 95% confidence intervals, and weights, with pooled estimates computed using a random effects model. Heterogeneity measures, prediction intervals, and subgroup analyses are also presented.

To evaluate the impact of MI on verbal communication in autism, we performed a meta-analysis by compiling data from six studies using various outcome measures, including the Peabody Picture Vocabulary Test–Fourth Edition (PPVT-4; this test, commonly used to assess verbal ability and language development, measures receptive vocabulary and verbal comprehension but does not directly evaluate expressive language), the Expressive One-Word Picture Vocabulary Test–Fourth Edition (EOWPVT-4), and the Conversational Communicative Scales (CCs) (Figure 4). The overall pooled analysis using a random-effects model yielded a non-significant effect (z = 0.66, *p*-value = 0.51), suggesting no consistent benefit of MI on verbal communication across the entire dataset. Importantly, there was substantial heterogeneity among the studies (I^2^ = 62%, τ^2^ = 0.04, *p*-value < 0.01), suggesting that true differences between study outcomes, beyond random sampling error, exist to a notable degree. A test for subgroup differences (χ^2^ = 15.83, df = 14, *p*-value = 0.32) was not statistically significant, but the result still indicated that the impact of MI may vary depending on the specific verbal communication outcome measured. While most subgroups showed no statistically significant changes, some specific tools, such as the Verbal Production Evaluation Scale (VPES), which focuses on the quantity and quality of verbal output, showed a moderately positive effect, though this result also did not reach statistical significance (z = 1.39, *p*-value = 0.16). Even more notably, the “Vowels” subgroup demonstrated a stronger positive effect, with a higher standardized MD, reaching statistical significance (z = 2.93, *p*-value < 0.01; I^2^ = 0%, τ^2^ = 0, *p*-value = 0.83). This suggests that MI may be particularly effective in enhancing phonemic-level expressive language abilities, such as vowel articulation, in autistic individuals.

This finding suggests that MIs may effectively enhance expressive language skills in autistic individuals, as measured by the VPES. Remarkably, the heterogeneity within this subgroup was moderate to high (I^2^ = 87%, τ^2^ = 1812.93, *p*-value < 0.01), indicating a relatively consistent effect across the included studies, despite variability. A further breakdown across the entire meta-analysis revealed that heterogeneity within individual subgroups ranged from negligible (I^2^ = 0%, τ^2^ = 0) to moderate (e.g., I^2^ = 67%, τ^2^ = 70.50), highlighting that even within outcome-specific analyses, variability remains. These variations may be due to differences in intervention design (e.g., active music-making *vs*. passive listening), duration, therapist involvement, or participant characteristics such as age, language level, and autism severity. Overall, while MI do not appear to produce a universal or robust effect on verbal communication in autism when averaged across all studies, certain subsets of interventions or outcome measures may hold promise. The considerable heterogeneity highlights the complexity of music’s role in therapeutic settings for autism, suggesting the need for more refined, targeted research designs and better harmonization of outcome measures in future studies.

##### 3.2.3.2 Non-verbal communication

Communication impairments are considered a key diagnostic characteristic of autism. Autistic individuals struggle with typical non-verbal communication methods such as facial expressions, gestures, body language, and maintaining eye contact, which can limit their overall communication skills (Thorsson et al., 2024; Stone et al., 1997). Music stands out as a fascinating, unique, and multi-faceted stimulus that engages our brain in processing different types of information simultaneously: visual, auditory, somatosensory, and motor. When individuals engage in music-making, they utilize these integrated sensory inputs to guide movement and expression (Schlaug et al., 2009). Since music-making activities might engage regions of the brain that overlap with regions that presumably contain mirror neurons (engaged by seeing, hearing, and doing an action), recent research suggests that music, particularly structured interventions such as rhythm-based exercises (LaGasse, 2014; Srinivasan et al., 2015; Yoo and Kim, 2018), interactive singing (Ozdemir et al., 2006), instrumental play (Meister et al., 2004), could provide a fun and effective way to boost non-verbal communication skills in autistic individuals. For instance, Sharda et al. (2018) showed that an 8- to 12-week improvisational intervention not only enhanced social communication but also improved the connections between auditory and motor brain regions in autistic children.

**Figure 5:**
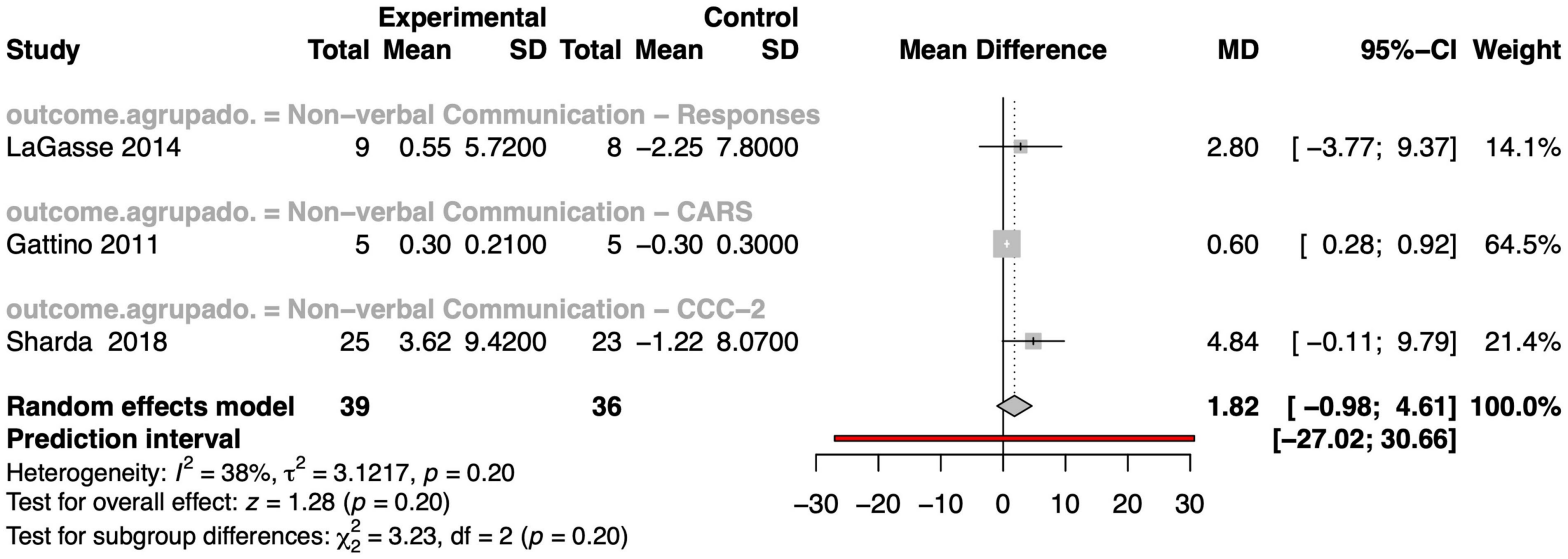
Forest plot displaying mean differences for non-verbal communication outcomes across studies involving autistic individuals. Results include individual study MDs, confidence intervals, and weights, with overall estimates calculated via a random effects model. The plot also reports heterogeneity statistics, prediction intervals, and significance tests for subgroups and overall effects.

Only three studies were eligible for inclusion in the meta-analysis evaluating the effect of MI on autistic individuals (Figure 5). The overall pooled effect size was small and statistically non-significant (MD = 1.82, 95% CI: −0.98 to 4.61, z = 1.28, *p*-value = 0.20), suggesting that MI did not lead to reliable improvements compared to control conditions. Heterogeneity was low (I^2^ = 38%, τ^2^ = 3.12, *p*-value = 0.20), indicating consistency across studies. The wide prediction interval (−27.02 to 30.66) suggests considerable uncertainty around the true effect under similar conditions. Among the included studies, Gattino et al. (2011) contributed the most weight (64.5%), and used the Childhood Autism Rating Scale (CARS), reporting a small but statistically significant mean difference (MD = 0.60, 95% CI: 0.28 to 0.92). The study by LaGasse (2014) reported a more favorable (MD 2.80), but a wide confidence interval (95% CI: −3.77 to 9.37). Similarly, Sharda et al. (2018) also reported a slight improvement using the CCC-2 (MD = 4.84, 95% CI: −0.11 to 9.79). The authors considered this test as a measure of pragmatic (social) communication, which in fact encompasses both verbal and non-verbal components. Nevertheless, this estimate is associated with considerable uncertainty.

Taken together, while the current meta-analytic evidence does not confirm a consistent or statistically significant effect of MI on non-verbal communication in autism, the presence of small positive trends, especially in studies using more sensitive or targeted measures, suggests potential. These findings highlight the need for further high-quality, large-scale studies to explore specific therapeutic conditions under which music may meaningfully benefit communication outcomes in autistic individuals.

#### 3.2.4 Quality of life

Quality of life (QoL) is an essential factor in understanding autism, as it includes physical, emotional, social, and psychological well-being. Autistic individuals often face challenges in communication, social engagements, sensory sensitivities, and mental health, which can lead to a lower QoL. Music has been explored as a therapeutic tool to enhance QoL by promoting emotional regulation, reducing anxiety, encouraging social connections, and offering a means of self-expression. Studies show that MI, such as MT and active music engagement, can enhance mood, boost social participation, and increase overall life satisfaction in autistic individuals (Sharda et al., 2018; Poquerusse et al., 2018).

Thompson et al. (2014) conducted a randomized controlled trial on family-centered music therapy for young children with severe autism, revealing notable improvements in social interactions both at home and in the community, as well as a strengthened parent–child bond. Similarly, Porter et al. (2017) explored the impact of music on children and autistic adolescente, finding intensifications in self-esteem and diminutions in depression scores, especially among participants aged 13 and older. Consequently, MI, particularly the active and engaging ones, can enhance social interaction and emotional well-being in autistic individuals, thereby positively impacting their QoL.

**Figure 6:**
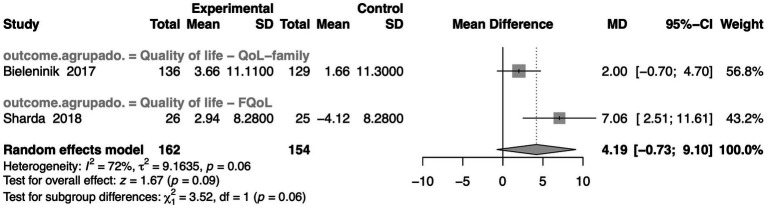
Forest plot presenting the effects of interventions on quality of life (QoL) in autistic individuals. The figure shows mean differences between experimental and control groups, confidence intervals, and study weights. Summary estimates were generated using a random effects model, and the figure includes heterogeneity measures, prediction intervals, and subgroup/overall effect tests.

Out of 120 articles reviewed, only two (Bieleninik et al., 2017; Sharda et al., 2018) met the criteria for our meta-analysis on the QoL in autistic individuals (Figure 6). The pooled effect size showed a positive trend favoring MI, with a MD of 4.19 [95% CI: −0.73 to 9.10], approaching statistical significance (z = 1.67, *p*-value = 0.09). This trend, while not definitive, suggests a meaningful potential for improvement in QoL through music-based therapies. Substantial heterogeneity was observed (I^2^ = 72%, τ^2^ = 9.16, *p*-value = 0.06), indicating considerable variability across studies, likely due to differences in outcome measures and participant characteristics, but also highlighting the diverse applicability of MI across contexts. Sharda et al. (2018) used the Family Quality of Life scale (FQoL), which is 25-item parent questionnaire assessing perceived support and family well-being, rather than the child’s QoL, and demonstrated a significant and robust improvement (MD = 7.06, 95% CI: 2.51 to 11.61), suggesting that MT may meaningfully enhance perceived QoL in certain contexts. Bieleninik et al. (2017) showed a modest mean effect (MD = 2.00), but with a wider confidence interval crossing zero (95% CI: −0.70 to 4.70), reflecting more variable results. Taken together, although the meta-analysis did not reach conventional statistical significance, the overall trend is encouraging. Particularly, the strong positive results from Sharda et al. (2018) support the view that MI can lead to meaningful improvements in QoL for autistic individuals, especially within family-oriented frameworks. These findings warrant further high-quality, targeted research and provide a promising foundation for incorporating MT into broader support strategies for the autistic community to improve QoL.

#### 3.2.5 Social interaction

Autistic individuals often face difficulties in social interaction, which can hinder their ability to build relationships and participate in shared activities. With the increasing interest in non-pharmacological interventions, MI have emerged as promising options for improving social skills in autistic individuals. The structured and predictable nature of music, along with its capacity to evoke emotional responses, makes it an effective medium for promoting social engagement. Subsequently, numerous studies have investigated the role of MI in improving social interaction in autistic individuals, providing evidence for their effectiveness in strengthening communication, emotional reciprocity, and social adaptation.

Bieleninik et al. (2017) explored the impact of MI on social interactions in autistic individuals using ADOS-SA and observed some social improvements, yet they were not significantly different from those in the standard care group. Their large-scale randomized controlled trial on MT did not demonstrate a clear advantage over standard care in social communication outcomes. In contrast, Ghasemtabar et al. (2015) found that MT enhanced social responsiveness and emotional engagement, supporting its role in fostering meaningful social interactions. Along with, another study (Kim et al., 2009) indicated that MT sessions improve social interaction skills in autistic children. This improvement is believed to stem from music’s ability to engage the brain’s reward systems and help regulate emotional responses, thus encouraging more positive social behaviors.

Supporting these findings, Porter et al. (2017) emphasized that interactive music sessions promote social bonding and turn-taking, essential components of social interaction. Additionally, Gattino et al. (2011) proved that MI led to notable improvements in social adaptation skills. Collectively, these studies suggest that MI can serve as a valuable therapeutic tool for enhancing social interaction in autistic individuals.

**Figure 7:**
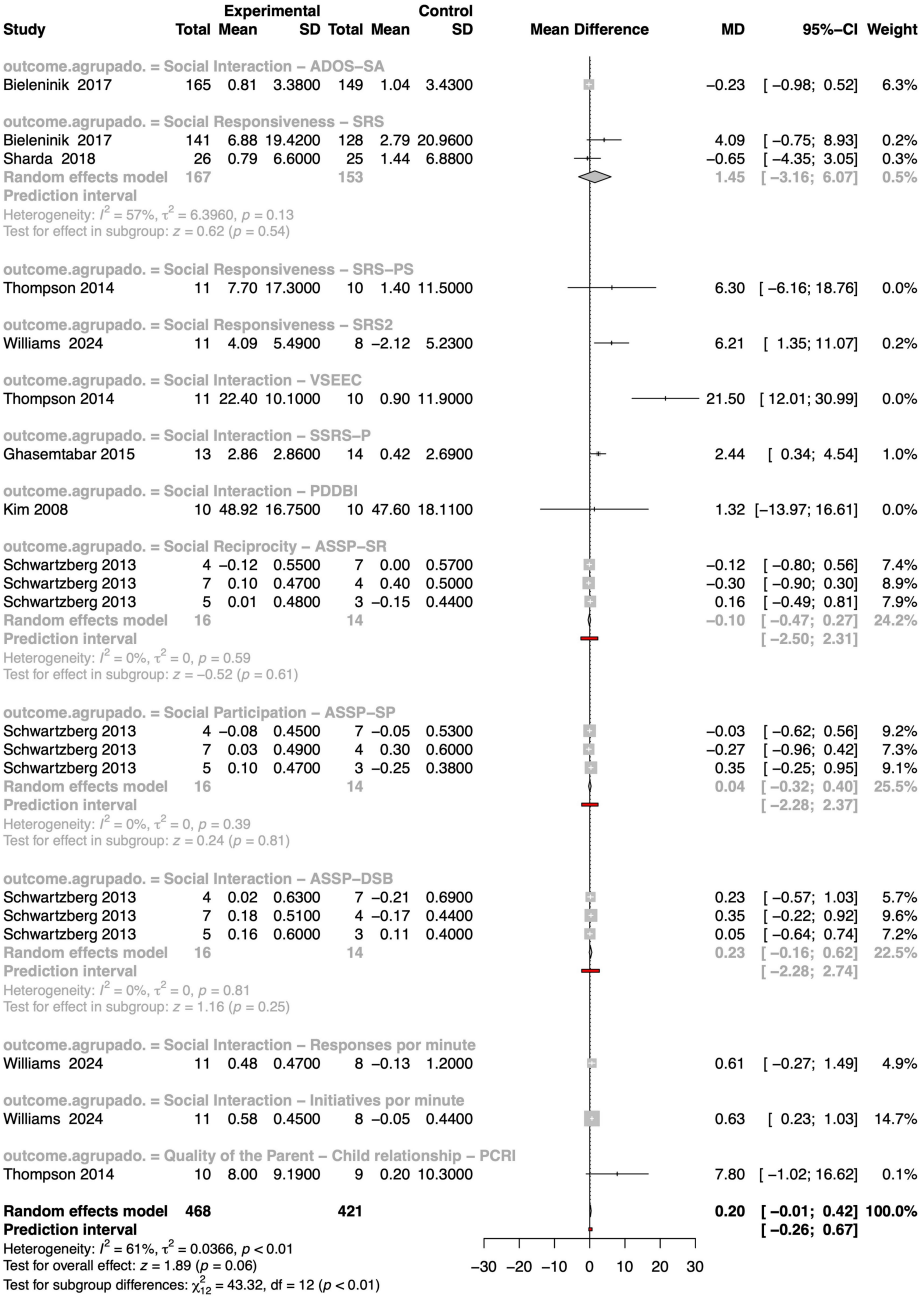
Forest plot presenting the effects of interventions on social interaction in autistic individuals. The figure shows mean differences between experimental and control groups, confidence intervals, and study weights. Summary estimates were generated using a random effects model, and the figure includes heterogeneity measures, prediction intervals, and subgroup/overall effect tests.

Out of the reviewed studies, seven met the inclusion criteria for our meta-analysis on social interaction outcomes in autistic individuals, most of which utilized the following scales: Autism Diagnosis Observation Schedule-Social Affect (ADOS-SA), Social Responsiveness Scale (SRS), and Autism Social Skills Profile (ASSP) (Figure 7). The pooled MD was 0.20, with a 95% confidence interval ranging from −0.01 to 0.42, approaching statistical significance (z = 1.89, *p*-value = 0.06). Although this result did not reach the conventional threshold for statistical significance, it indicates a small but consistent positive effect of MI on social interaction skills. The presence of moderate heterogeneity (I^2^ = 61%, τ^2^ = 0.04, *p*-value < 0.01) reflects differences in study populations, interventions, and, notably, outcome measurements. Most importantly, a significant test for subgroup differences (χ^2^ = 43.32, df = 12, *p*-value < 0.01) revealed that the observed effects varied meaningfully across the different types of social interaction outcomes assessed.

The studies encompassed a wide range of validated tools, each targeting distinct dimensions of social functioning. The ADOS-SA subgroup used the Autism Diagnostic Observation Schedule, Social Affect domain, which captures clinician-observed core deficits in social communication. In contrast, the Social Responsiveness – SRS, SRS-PS (preschool version), and SRS-2 subgroups relied on the Social Responsiveness Scale in its various forms, a widely adopted parent- or teacher-report instrument designed to measure the severity of autism-related social difficulties in everyday life. Other subgroups focused on different developmental and relational aspects of social interaction. For example, the VSEEC group used the Vineland Social–Emotional Early Childhood Scales to assess emotional and social functioning in young children, while the SSRS-P subgroup employed the Social Skills Rating System – Parent form to evaluate caregiver perceptions of social behavior. The Pervasive Developmental Disorders Behavior Inventory (PDDBI) offers a broader behavioral assessment that includes communication and adaptive functioning. Several subgroups drew on subdomains of the Assessment of Social and Sensory Participation (ASSP). These included Social Reciprocity – ASSP-SR, which reflects mutual, responsive social behaviors; Social Participation – ASSP-SP, capturing engagement in structured and unstructured social contexts; and ASSP-DSB, which focuses on daily social behavior. In addition to these standardized assessments, some studies measured more granular behavioral outcomes such as “Responses per minute” and “Initiatives per minute,” derived from observational coding of spontaneous social behaviors in naturalistic settings. Lastly, the Parent–Child Relationship – PCRI subgroup used the Parent–Child Relationship Inventory to assess perceived relational quality, including communication and emotional closeness between caregiver and child.

Among the various subgroups analyzed, only three demonstrated statistically significant improvements in social interaction outcomes following MI. The VSEEC subgroup showed the most substantial effect, with a MD of 21.50 and a 95% CI of [12.01; 30.99], indicating a large and robust improvement in social–emotional functioning in young children. Similarly, the SRS2 subgroup showed a significant positive effect (MD = 6.21, 95% CI: [1.35; 11.07]). Additionally, the SSRS-P subgroup revealed a moderate but meaningful improvement (MD = 2.44, 95% CI: [0.34; 4.54]), suggesting increased caregiver-observed social engagement.

Taken together, these results are promising. Although the overall meta-analytic effect narrowly missed statistical significance, the consistent positive direction of the effects, combined with substantial and meaningful subgroup differences, indicates that MI can foster improvements in social interaction among autistic individuals, particularly when targeted to specific contexts and measured through responsive, ecologically valid tools. These findings emphasize the importance of selecting appropriate outcome measures and tailoring interventions to individual needs, and they justify further high-quality, stratified research in this area.

## 4 Discussion

Research on musical processing and abilities in autistic individuals reveals a distinctive cognitive profile, often described as a potential “musical phenotype.” Among 45 studies, findings consistently show that many autistic individuals, even without formal musical training, demonstrate enhanced sensitivity to pitch changes, superior pitch discrimination, and in some cases, a higher prevalence of absolute pitch compared to the general population. These abilities appear to stem from atypical cognitive styles, including heightened perceptual functioning, focused attention, and in some cases, superior memory for musical material. Although musical processing in autism is often preserved, certain aspects, such as pitch production or emotional expressivity, can vary, with some impairments emerging particularly in speech contexts. Emotional responses to music in autism are generally comparable to those of TD individuals, though differences in physiological arousal, processing speed, and neural activation patterns suggest unique underlying mechanisms. Neuroimaging studies further support these distinctions, indicating that while basic emotional recognition remains intact, brain regions involved in timing, coordination, and autonomic regulation may function differently during musical experiences in autism (Gebauer et al., 2014). Studies also show that autistic individuals experience strong preferences for certain types of music, with preferences shaped by both sensory sensitivity and therapeutic utility. For instance, simpler musical structures (e.g., predictable rhythms and consonant harmonies) appear particularly effective in promoting attention and reducing stereotypical behaviors, especially in individuals with more severe autism symptoms (Sharda et al., 2018; Caria et al., 2011). Qualitative research emphasizes the importance of music in emotional self-regulation, identity formation, and social connection, reinforcing its role as a meaningful and supportive element in the lives of autistic individuals. Collectively, these results highlight music’s potential as a powerful tool for engagement, communication, and personal expression within this population.

Our investigation aimed to evaluate the effectiveness of MI in autistic populations. The synthesis encompassed a wide range of outcomes, including attention, verbal and non-verbal communication, QoL, behavior, and social interaction, assessed using various standardized measures. The findings reveal a complex pattern of results, with some domains exhibiting promising effects and others showing limited or non-significant improvements. Notably, the heterogeneity observed both across and within outcome domains reflects variability in study designs, intervention types, measurement tools, and participant characteristics. Overall, the results suggest that MI has a moderate, positive effect on several developmental outcomes in autistic individuals, supporting its value as a therapeutic approach. The results of our meta-analysis provide a noteworthy contribution when compared to prior systematic reviews in the field of autism and MI. This is primarily due to the more rigorous methodological framework we applied, which included clearly defined inclusion and exclusion criteria that carefully considered participant characteristics, intervention types, and study design. Our review also encompassed a broader range of participant ages, timeframes, and outcome domains, enhancing the comprehensiveness and applicability of the findings. Crucially, we combined both qualitative and quantitative analytical approaches, which allowed for a more balanced and context-sensitive interpretation of results, particularly given the diversity of outcome measures used across studies. By focusing on different outcomes that hold direct relevance for autistic individuals, this work adds meaningful depth to the existing body of literature.

A total of 15 trials met the criteria for inclusion in the meta-analysis, evaluating the effects of MI on autistic individuals ranging from two years of age to young adulthood. These interventions were compared against standard care or placebo-like control therapies designed to isolate the unique contribution of music itself by controlling for non-specific factors such as therapist attention and participant engagement. Our meta-analyses aimed to evaluate the effectiveness of MI, including music-making, receptive music listening, and MT, across several developmental domains in autistic individuals, including attention, behavior, verbal and non-verbal communication, QoL, and social interaction. Overall, the findings present a complex picture: while statistically significant effects were not consistently observed across all domains, multiple positive trends emerged, particularly within specific subgroups and outcome measures (Sa, 2020; Williams et al., 2024; Srinivasan et al., 2015; Chenausky et al., 2022). These results highlight both the therapeutic potential of MI, and the complexity involved in evaluating its efficacy in autistic populations. The findings align with theoretical perspectives suggesting that music may engage neural systems associated with emotion, language, and social cognition, domains that are typically affected in autistic individuals (Lai et al., 2012).

Across the attention domain, the pooled results did not yield statistically significant effects. However, a small positive trend was observed, particularly after removing a single outlier from the analysis, which strengthened the results and suggests that music may modestly influence attentional processes. High heterogeneity across the included studies highlights the variation in measurement tools, intervention formats, and participant characteristics, suggesting that the potential for MI to enhance attention may depend heavily on the type of attentional skill targeted and the contextual features of the intervention. Given that musical experiences often require sustained focus and dynamic engagement, further exploration is warranted, particularly with refined methodologies and attention subtypes more closely aligned with musical structures. Behavioral outcomes showed no significant overall effect, although individual studies yielded mixed findings. Some reported meaningful improvements, while others observed null or inconsistent effects. Notably, removing a single outlier from the meta-analysis resulted in a more consistently positive outcome, reaching marginal statistical significance. The extremely high heterogeneity across studies, driven by differences in behavioral scales, intervention types, and participant characteristics, suggests that the broad category of ‘behavior’ may be too general to capture the specific domains in which MI might be most effective. Rather than expecting uniform behavioral changes, future research should focus on targeted areas such as emotional regulation, repetitive behaviors, or adaptive functioning, using standardized and validated assessment tools. In terms of verbal communication, the pooled analysis across various outcome measures again failed to reveal a statistically significant effect. However, a closer examination of subgroups uncovered more promising findings. Notably, MI appeared to enhance expressive language abilities, particularly at the phonemic level, such as vowel articulation, where the effects reached statistical significance. These findings are consistent with the natural overlap between musical and linguistic processing, especially regarding rhythm, pitch, and prosody. The capacity of music to structure and scaffold auditory input may thus support specific speech production skills in autistic individuals. This domain exemplifies the importance of fine-grained outcome analysis, as global language measures may obscure more focal gains. The analysis of non-verbal communication outcomes did not yield significant effects either, though small positive trends were observed. Interestingly, heterogeneity in this domain was relatively low, which may suggest a more consistent, albeit modest, impact of MI. Individual studies employing sensitive observational tools reported slight improvements, indicating that MI might influence non-verbal communicative behaviors such as gestures, eye contact, or joint attention, particularly when interventions are developmentally attuned and interaction-focused. In contrast, the results related to QoL were more encouraging, despite being drawn from only two studies. The pooled effect approached statistical significance, and one study demonstrated a robust improvement in family-related QoL outcomes. This points to the broader psychosocial benefits of MT, which may extend beyond individual-level symptom change to influence familial and relational well-being. These findings support a shift toward evaluating MI not solely on clinical symptoms, but on broader, person-centered outcomes that reflect real-world functioning and QoL. Among all domains, social interaction showed the most consistently positive pattern. Although the overall effect narrowly missed statistical significance, multiple subgroups, especially those relying on caregiver reports or ecologically valid observational tools, demonstrated significant improvements. Instruments such as the VSEEC, the SRS-2, and the SSRS-P captured meaningful changes in social behaviors, suggesting that MI may enhance social engagement, reciprocity, and functional interaction in everyday contexts.

Our findings seem to support the notion that MI outperforms standard care and has therapeutic properties. The musical experience itself, especially when grounded in relational dynamics and shaped by the individual’s interests and motivations, appears to foster social engagement in ways that more traditional therapies may not. These approaches have shown promise in enhancing core social communication skills, such as initiating interactions, maintaining eye contact, and interpreting emotional stimuli (Gattino et al., 2011).

Despite the promising outcomes identified in this review, several methodological and conceptual limitations should be acknowledged. A major restriction lies in the relatively small sample sizes of many included studies, which diminish statistical power and limit the generalizability of the findings across the heterogeneous autism spectrum. Future research would greatly benefit from larger, more representative cohorts that account for variability in age, language ability, and cognitive profiles. Additionally, most studies investigated only short-term effects, making it difficult to assess whether observed gains in communication, attention, behavior, or QoL are sustained over time. The lack of longitudinal follow-up data hinders our understanding of the long-term efficacy and developmental trajectory of MI, which is particularly relevant in neurodevelopmental conditions such as autism. Another key limitation concerns the ecological validity of the outcome measures used to evaluate the impact of MI. As highlighted by Heaton et al. (2007), musical assessments often rely on controlled tasks that do not adequately capture the complexity of real-world musical experiences. Some experimental paradigms focus narrowly on isolated musical features, such as pitch or rhythm discrimination, without considering how music is experienced dynamically, socially, and emotionally in everyday life. This methodological gap may lead to underestimations of music’s broader impact on engagement, emotional regulation, and social connection. Moreover, standardized tests may fail to reflect individual preferences, sensitivity, and the highly personalized nature of musical engagement in autistic individuals (Korošec et al., 2024).

Therefore, to advance the field, future research should prioritize longitudinal designs that can track outcomes over extended periods and across real-world settings. There is also a need for greater attention to the ecological validity of outcome measures, incorporating assessments that capture spontaneous musical interaction and meaningful engagement. Understanding the specific musical abilities and processing profiles of autistic individuals is essential for developing neurobiologically grounded, personalized interventions. Moreover, the incorporation of biological data, such as genetic, epigenetic, or transcriptomic information, into intervention studies [in line with other disease conditions, see Gómez-Carballa et al. (2023), Navarro et al. (2023), and Navarro et al. (2021)] could provide a deeper understanding of the underlying mechanisms and help identify biomarkers predictive of treatment response. Integrating behavioral outcomes with molecular and neurophysiological measures represents a promising direction for future research (Latif et al., 2021).

Despite the well-documented difficulties that autistic individuals may encounter when processing complex emotional or social indicators, numerous studies have demonstrated that the recognition of basic emotions in music is often preserved. This suggests that music constitutes a domain of both relative cognitive strength and powerful intrinsic interest. As a non-verbal, emotionally resonant medium, music offers an alternative channel of communication that is particularly valuable in populations with language impairments.

Taken together, the findings of this meta-analysis suggest that while MI may not yield uniformly large or statistically robust effects across all developmental domains in autism, they show meaningful promise when applied in a targeted, individualized, and context-sensitive manner. The considerable heterogeneity across studies, stemming from differences in intervention formats (e.g., active *vs*. passive engagement, session length, therapist involvement), participant characteristics (e.g., age, language ability, autism severity), and outcome measures complicates broad generalizations but also highlights the flexibility of MI to meet diverse needs. Notably, emerging patterns of benefit in areas such as expressive language, phonemic articulation, and social interaction point to the promising capacity of music to promote engagement and foster meaningful connection. As an empirically grounded and neurodiversity-affirming tool, MI represents a compelling complement to conventional therapeutic approaches. Future research should explore long-term effects, skill generalization, and the role of individual differences in treatment response to better integrate MI into personalized, responsive, and holistic care for autistic individuals.

## Data Availability

The original contributions presented in the study are included in the article/Supplementary material, further inquiries can be directed to the corresponding author.

## Footnotes

^1^ https://www.bmj.com/content/372/bmj.n71

## Supplementary material

SUPPLEMENTARY FIGURE S1 Forest plot illustrating the effect sizes (mean differences) for studies comparing intervention and control groups on attention outcomes in autistic individuals, excluding the study by Sa (2020) from the meta-analysis.

SUPPLEMENTARY FIGURE S2 Forest plot illustrating the effect sizes (mean differences) for studies comparing intervention and control groups on attention outcomes in autistic individuals, excluding the RBS-R Sensory subscale results from Srinivasan et al. (2015) from the meta-analysis.
