# Supplementary figures and images for: The effect of music interventions in autism spectrum disorder: a systematic review and meta-analysis
Source: Front Integr Neurosci. 2025 Oct 28;19:1673618. doi: 10.3389/fnint.2025.1673618 (PMC12602440; doi:10.3389/fnint.2025.1673618)

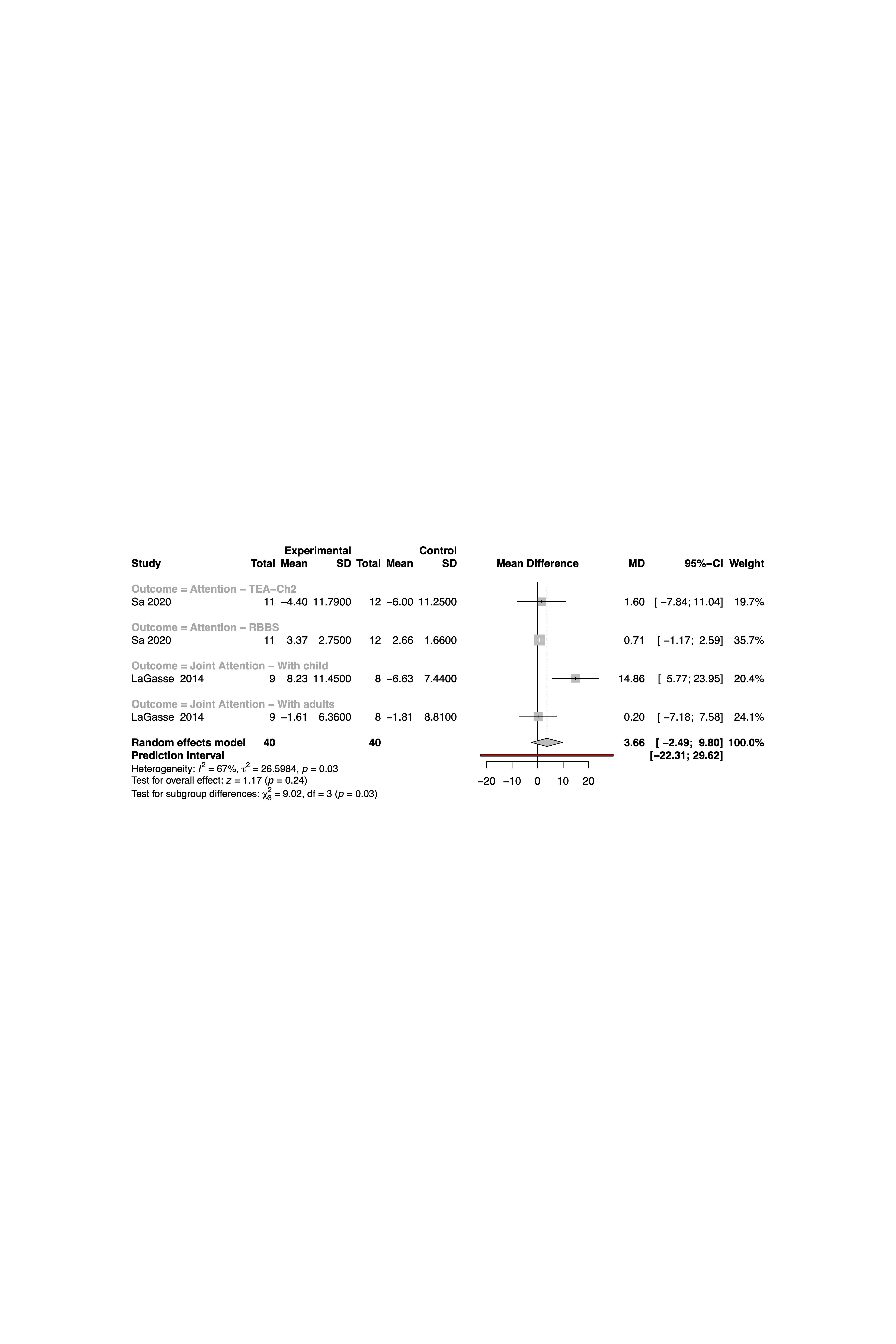

Supplement: SUPPLEMENTARY FIGURE S1 — Forest plot illustrating the effect sizes (mean differences) for studies comparing intervention and control groups on attention outcomes in autistic individuals, excluding the study by Sa (2020) from the meta-analysis. [file Image_1.JPEG]

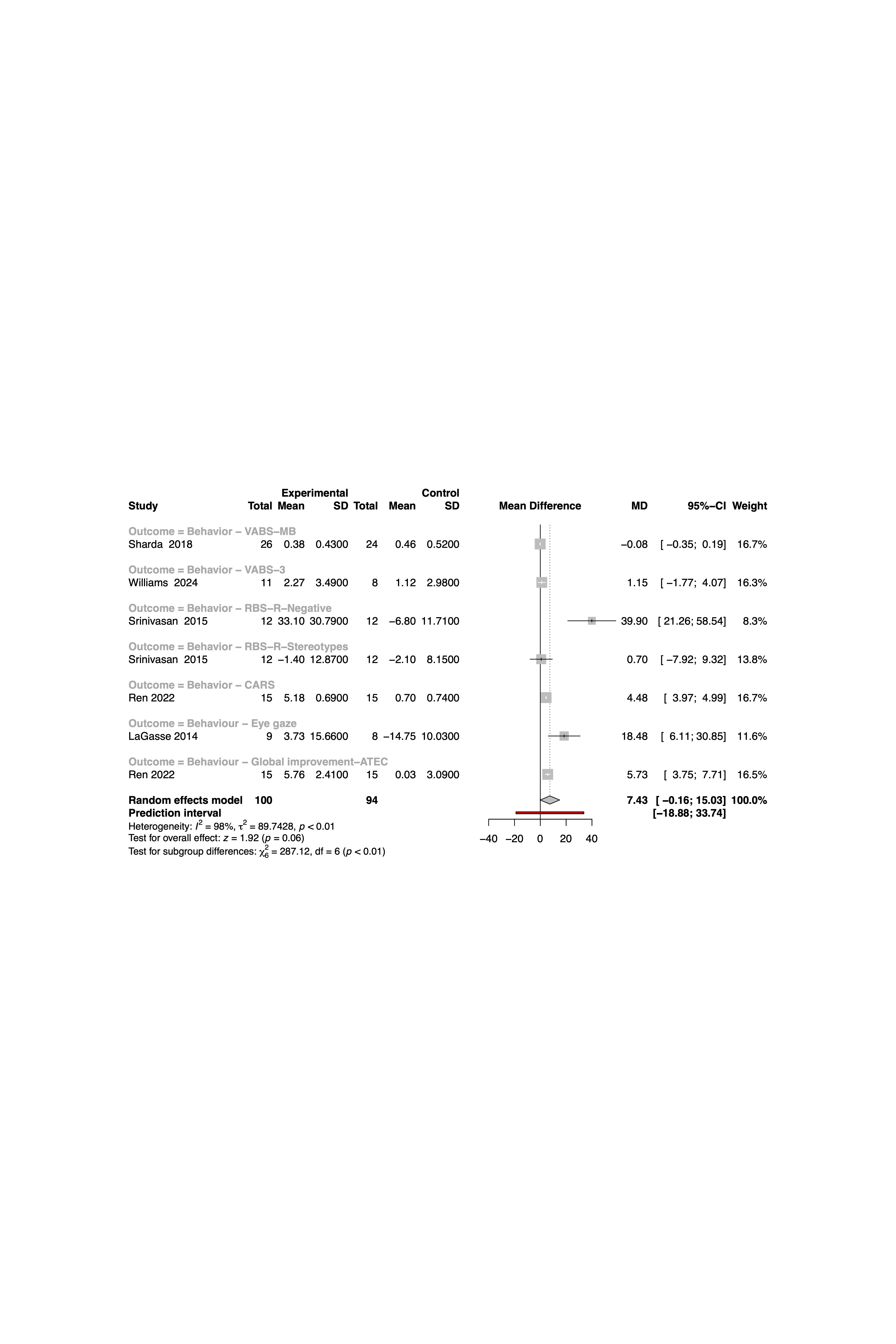

Supplement: SUPPLEMENTARY FIGURE S2 — Forest plot illustrating the effect sizes (mean differences) for studies comparing intervention and control groups on attention outcomes in autistic individuals, excluding the RBS-R Sensory subscale results from Srinivasan et al. (2015) from the meta-analysis. [file Image_2.JPEG]
